# Nanosilver Particle Production Using *Juglans Regia* L. (Walnut) Leaf Extract

**Published:** 2013-02-13

**Authors:** Hassan Korbekandi, Gholamreza Asghari, Susan Sadat Jalayer, Maryam Sadat Jalayer, Maedeh Bandegani

**Affiliations:** 1 Department of Genetics & Molecular Biology, School of Medicine, Isfahan University of Medical Sciences, Isfahan, IR Iran; 2 Isfahan Pharmaceutical Sciences Research Center, School of Pharmacy, Isfahan University of Medical Sciences, Isfahan, IR Iran; 3 Department of Biotechnology, Karaj Payam Noor University, Karaj, IR Iran; 4 Department of Science, Tarbiat Modares University, Tehran, IR Iran

**Keywords:** Silver, Nanoparticles, Biosynthesis, Plant Extract

## Abstract

**Background:**

The production of nanoparticles using a biosystem is considered green chemistry. Application of plant extracts as a biological process has been proven to be suitable for synthesis of nanoparticles.

**Objectives:**

This study designed in order to evaluate the production of silver nanoparticles using *Juglans regia* leaf extract and to compare the outcome of different preparation methods of plant extracts (ethanolic extract, boiling water extract and plant powder) for the generation of nanoparticles.

**Materials and Methods:**

The reaction mixture contained the following ingredients: AgNO_3_ (10 mM) as the biotransformation substrate, plant extract or powder as the biocatalyst, glucose (560 mM) as the electron donor, phosphate buffer (pH = 7, 100 mM) and ethanol 70% as the solvent in the reaction mixture. The samples were taken from the reaction mixtures at different times, and the absorbance (450 nm) of the colloidal suspensions of silver nanoparticle hydrosols was recorded immediately following dilution (1:80) so as to preserve its freshness.

**Results:**

UV-visible spectrophotometer analysis revealed that the direct application of powder of the walnut leaf was the most efficient technique. TEM (Transmission electron microscopy) micrograph obtained by using this method revealed the generation of aggregated polydisperse, quasi-spherical nanoparticles in sizes of 10-50 nm. Ethonolic extract resulted in single silver nanoparticles which were nearly monodisperse, spherical, and individual nanoparticles ranged in size from 1-5 nm. Therefore, using direct powder of Walnut created more particles but applying ethanolic extract synthesized particles with smaller dimensions and no aggregation.

**Conclusions:**

Different preparation methods of *Juglans regia* influence silver nanoparticles formation.

## 1. Background

One of the important areas of research in nanotechnology is investigating the production of nanoparticles (NPs) of different sizes, shapes and controlled dispersion (1). Currently, nanometal particles have gained significant attention (particularly silver), due to their broad uses in the areas of electronics, material science and medicine (2). Unfortunately, most of the synthetic methods are expensive, toxic and not ecofriendly (3), and thus there is an ever growing need for green chemistry to use biosystems for production of nanoparticles (4). Application of plant extracts is the preferred method over other biological processes, because the complex process of maintaining cell cultures are removed in this technique and it is also suitable for large-scale synthesis of nanoparticles (5). So far many reports have been published in literature on the biogenesis of silver NPs using several plant extracts (6-32).

## 2. Objectives

In this study, green synthesis of Ag NPs using *Juglans regia* leaf without the presence of hazardous and toxic solvents and wastes were investigated.

## 3. Material and Methods

*Juglans regia* L. and *Camellia sinensis* L. (a previously proven plant as positive control) leaves were freshly obtained from Isfahan Agricultural and Natural Resources Research Center. Extractions were done following washing, air drying at room temperature and powdering the plant. Ethanolic extract was prepared by percolation (48 h), yielding 100 g plant powder extracted with hydro alcoholic solution (70%, 500 mL) using a 2 L percolator. The extract was concentrated to 50 mL. To prepare boiling water extract, 20 g plant leaf powder was boiled with 100 mL HPLC grade water (15 minutes). After filtering (Watman No: 4), clear extract was obtained (21).

The reaction mixtures contained (final concentration): AgNO_3_ (1 mM) as the substrate, glucose (560 Mm) as the electron donor, phosphate buffer (pH =7, 25 mM), plant extract or powder as the biocatalyst and ethanol 70% as the solvent in reaction mixture. The aforementioned ingredients were added in appropriate volumes into Duran® bottles (100 mL) and were incubated and shaken (70 rpm) at room temperature. Samples (1.5 mL) were taken from the reaction mixtures at different times, and the absorbance (450 nm) of the colloidal suspensions of silver nanoparticles (hydrosols) was read freshly (without freezing) and immediately after dilution (1:80). The absorption spectra were measured on a Shimadzu (UV mini-1240) spectrophotometer. The UV/Vis absorption spectrum of colloidal Ag was examined to monitor the bioproduction of silver nanoparticles. The ultraviolet visible absorption spectra of samples were evaluated at different time intervals after the start of the reaction. The λmax was about 450 nm in all samples. Transmission electron microscopy (TEM) was performed on selected samples in order to investigate the process of formation of silver nanoparticles and to study their size and shape. Samples for TEM were prepared by drop-coating the Ag nanoparticle solutions on to carbon-coated copper grids. Micrographs were obtained using an EM 900 ZEISS transmission electron microscope.

## 4. Results

### 4.1. Visual Observation

When plant extract or powder was exposed to Ag+ ions (AgNO_3_, 1 mM), the color of the reaction mixture turned yellowish brown, and then dark brown, which was consistent with the former studies, and was considered as the production of colloidal suspension (hydrosol) of silver nanoparticles (10, 21, 30). The appearance of dark brown is due to excitation of surface plasmon resonance in the nanoparticles.

### 4.2. Monitoring Synthesis of Silver Nanoparticles

**Figure 1:**
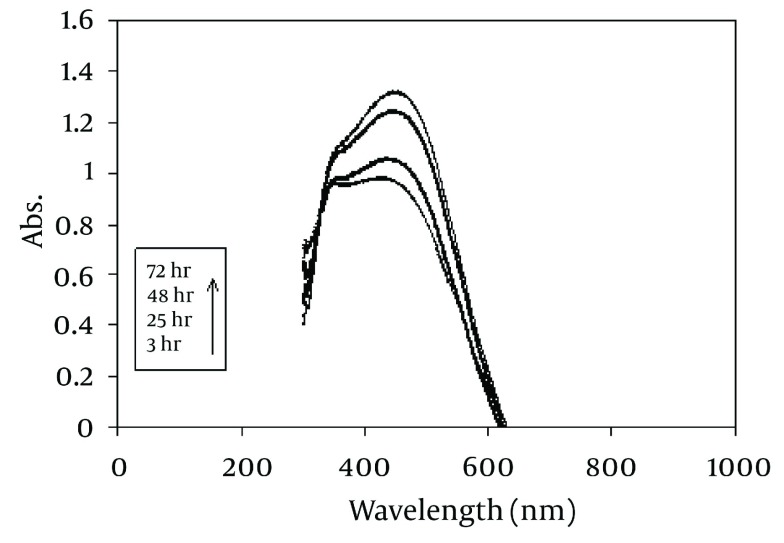
UV/V is Absorption Spectrum of the Produced Colloidal Ag Using *J. regia*

During the reaction period (from 3 h to 72 h), an increase in absorbance was observed in this wavelength, which is likely due to the increase in the production of colloidal silver nanoparticles ( Figure 1 ). Thus, the absorption spectrums of Ag hydrosols obtained in previous studies were confirmed (10, 26).

### 4.3. Observations Regarding Preparation of Samples for Analysis

In the samples, separation of biomass eliminated the interference in reading UV/V is absorbance (450 nm) of colloidal silver nanoparticles. It was assumed that sample filtration with filter discs (0.2 µm) or by centrifugation (15294 g, Eppendorf centrifuge 5417 R) could be useful. However after using both of these methods, the absorbance (450 nm) of colloidal suspension of nanoparticles decreased greatly to near zero and the color (yellow to brown) disappeared. This might be due to the aggregation of nanoparticles or absorption of them in the biomass. Therefore, filtering and centrifuging the samples revealed negative effects on nanoparticles and should be avoided. Instead of these two methods, samples were diluted (dilution factor = 80) and their absorbance was recorded at 450 nm.

### 4.4. Nanosilver Particle Formation by Ethanolic Extract

**Figure 2:**
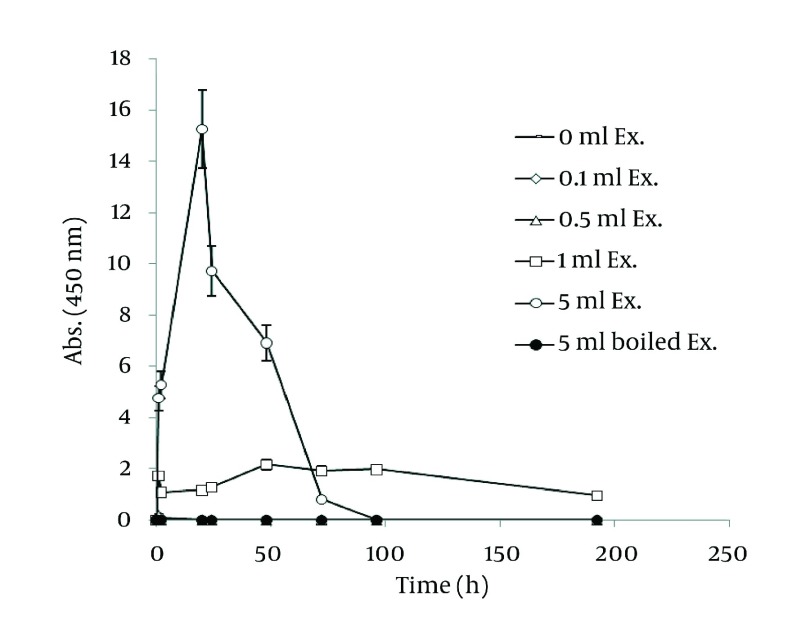
Time Course of Bioformation of AgNO_3_ Using Different Concentration of *J. regia* Ethanolic Extract

**Figure 3:**
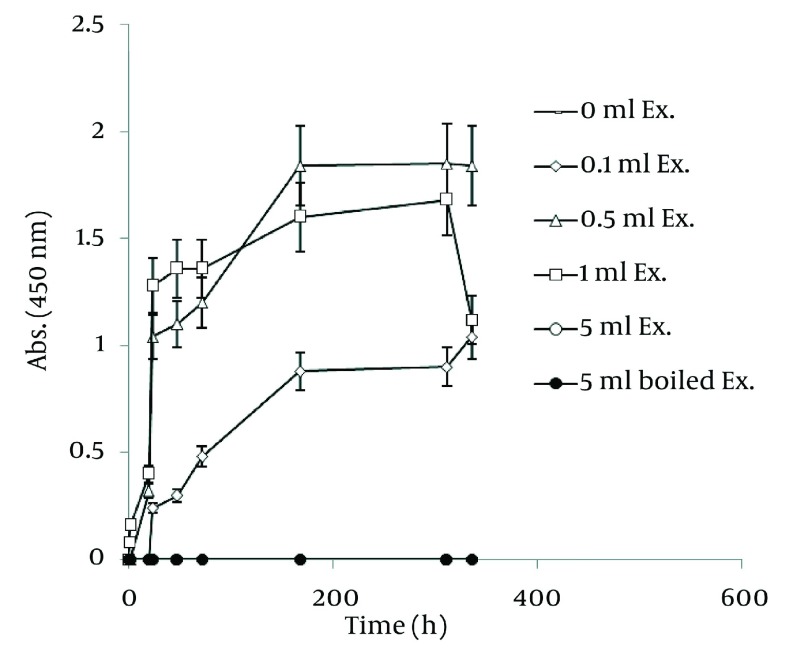
Time Course of Bioformation of AgNO_3_ Using Different Concentration of *C. sinensis* Ethanolic Extract

In order to extract plant phytochemicals, ethanol (70%) is a good solvent as it can dissolve and extricate biomolecules well and percolation is the most suitable technique to handle extraction process. Figure 2 reveals the time durations of silver nanoparticle formation with different walnut leaf ethanolic extract concentrations. By increasing the extract concentration, the number of produced nanoparticles was increased. The highest production of silver nanoparticles was observed at volume of 5 mL, 2 h after reaction initiation and then gradually decreased. In the case of boiled ethanolic extract, no absorbance was observed. It might be due to the destruction of reductive agents in the extract by boiling. In case of *C. sinensis*, the reaction rate was lower and increased to 168 h, and then nearly fixed ( Figure 3 ). It appears that the stability of these nanoparticles were better than those produced by walnut. The maximum absorption of Ag nanoparticles occurred at 0.5 mL and higher concentrations of extract cut down the absorption. It might be interpreted that this concentration of extracts had negative effects on synthesis process. The boiled ethanolic extract had also no absorbance, which could be caused by the same reason as previously explained.

**Figure 4:**
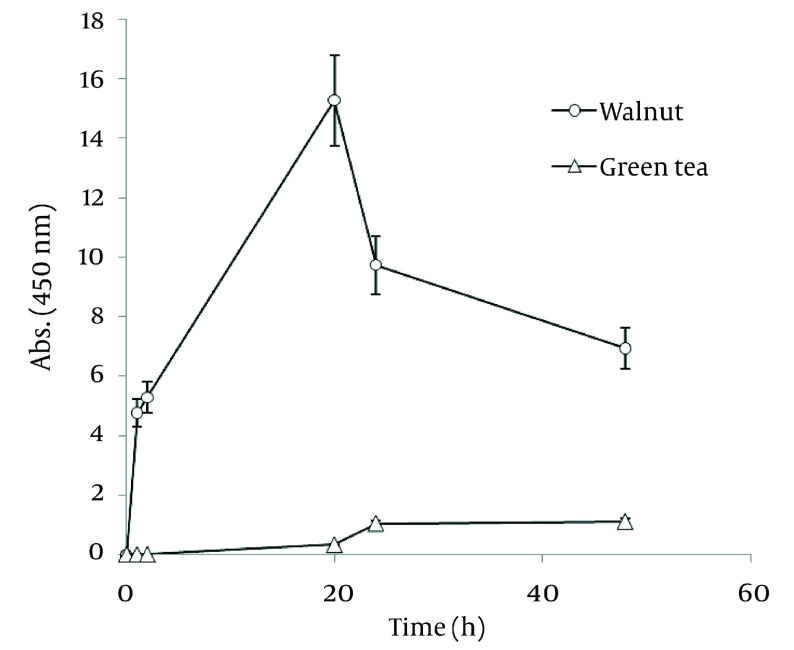
Comparison of Ag Nanoparticle Formation of Ethanolic Extracts of Walnut and Green tea

In order to compare nanoparticle production capability of the examined plants, the best results of their absorbance spectra has been revealed in Figure 4. According to the graph, *J. regia* has a much higher potential in nanoparticle synthesis than *C. sinensis*.

### 4.5. Nanosilver Particle Formation by Boiling Water Extract

**Figure 5:**
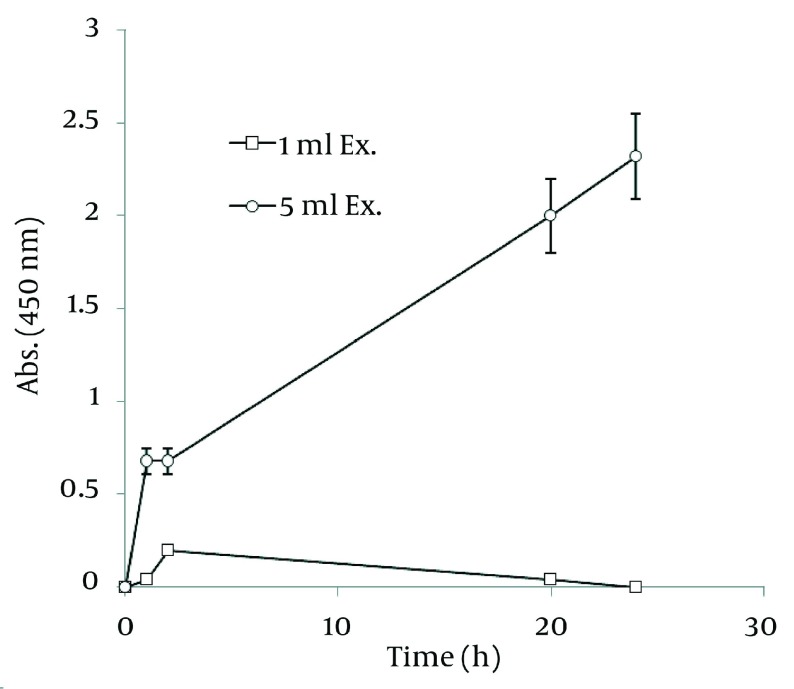
Time Course of Bioformation of AgNO_3_ Using Different Concentration of *J. regia* Boiling Water Extract

**Figure 6:**
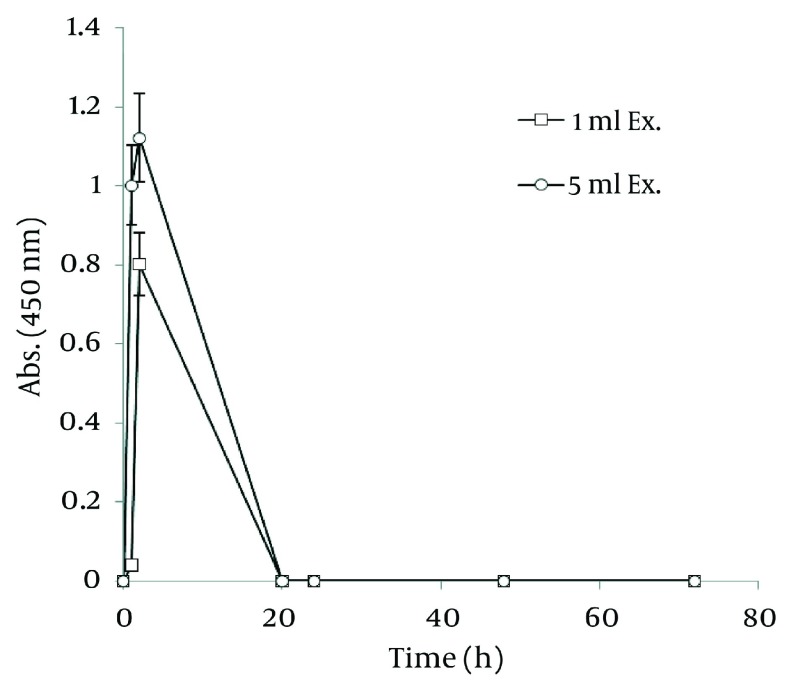
Time Course of Bioformation of AgNO_3_ Using Different Concentration of *C. sinensis* Water Extract

**Figure 7:**
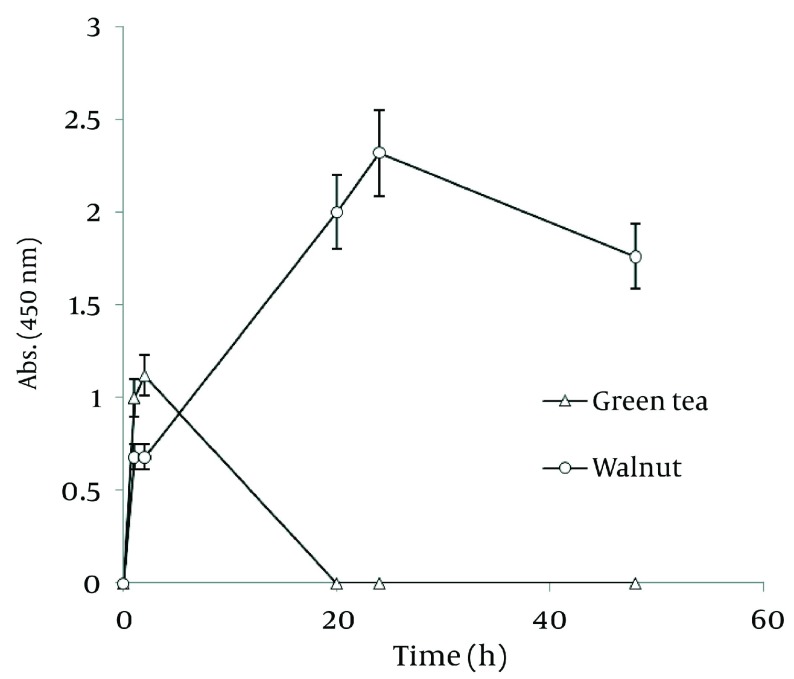
Comparison of Ag Nanoparticle Formation of Water Extracts of Walnut and Green tea

To investigate the impact of plant boiling water extract (which has been used in many previous studies) (5-7, 9, 11, 21) upon nanoparticle production, different concentrations of Walnut leaf boiled water extract were studied. The absorbance in the reaction mixture with highest amount of extract increased to 24 h ( Figure 5 ). At a lower concentration, the absorbance was lower and declined after 2 h. In the case of *C. sinensis*, the reaction rate was faster than Walnut and increased during the first hour, then quickly reduced. In the reaction mixture with more extract concentration, the absorbance was higher ( Figure 6 ). By comparing the best absorbance spectra peaks of using two plant boiling water extracts, again the amount of nanoparticle production was very high in the case of Walnut, but *C. sinensis* had a faster reduction rate ( Figure 7 ).

### 4.6. Nanosilver Particle Formation by Plant Powder

In former studies, the powder of the dried plant was directly located in the substrate solution for producing metal nanoparticles (23, 33). Therefore, the effect of this method on nanoparticle synthesis should be evaluated as well.

**Figure 8:**
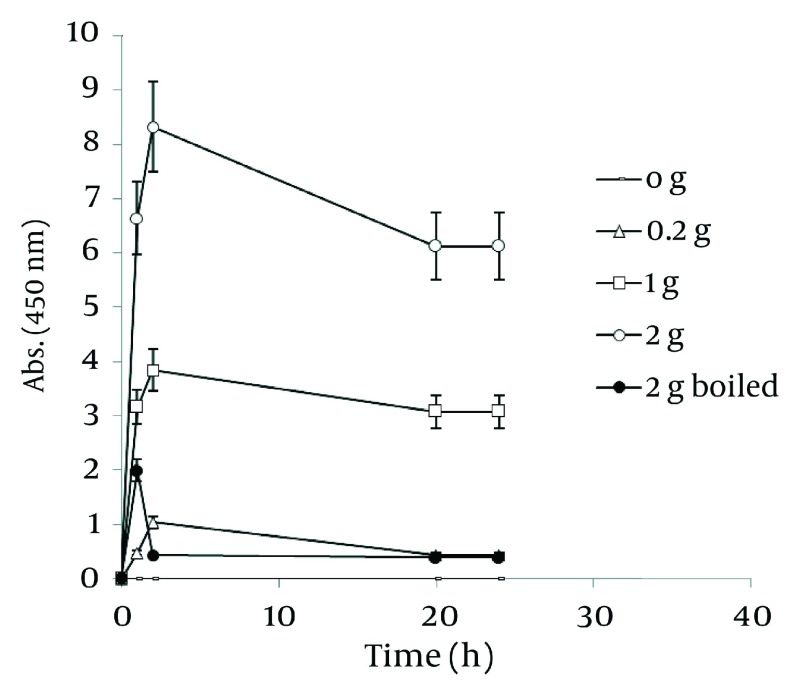
Time Course of Bioformation of AgNO_3_ Using Different Amount of *J regia* Powder

**Figure 9:**
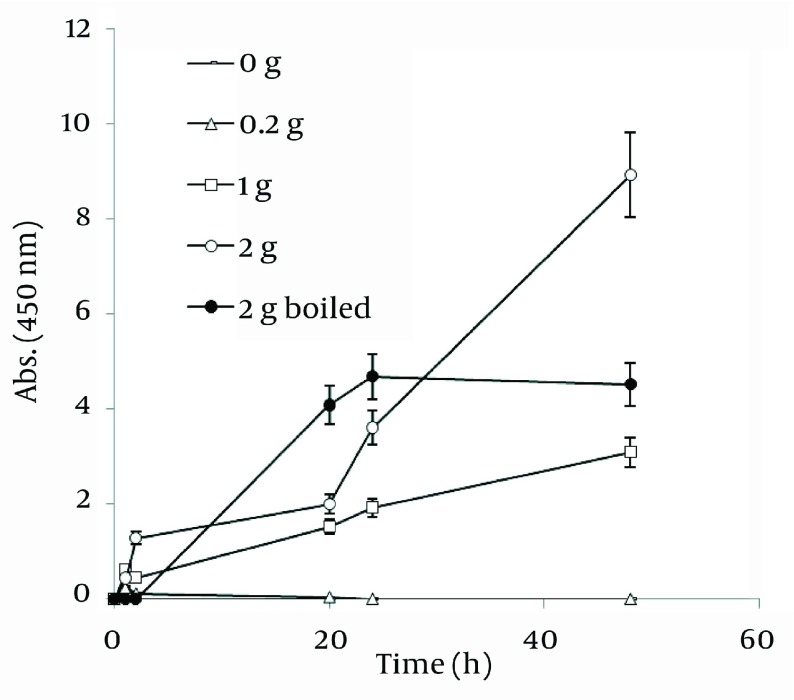
Time Course of Bioformation of AgNO_3_ Using Different Amount of *C. sinensis* Powder

**Figure 10:**
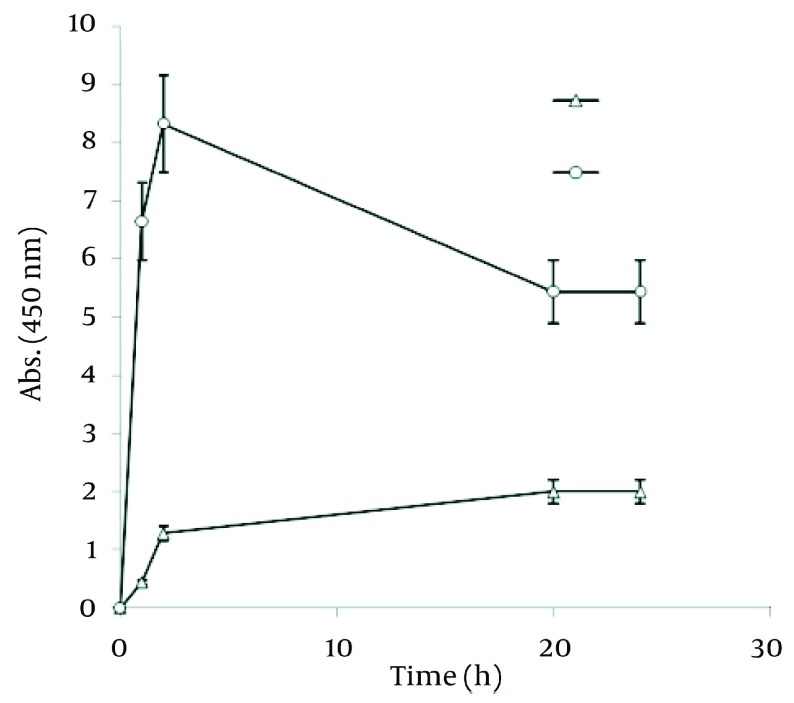
Comparison of Ag nanoparticle formation of Walnut and Green tea powder

Figure 8 shows a straight relationship between the amount of Walnut leaf powder and Ag nanoparticle productivity. The absorbance intensified in the second hour and then decreased. The kinetic energy of produced nanoparticles by using several different powder amounts were the same. Boiling the resultant mixture containing the plant powder caused considerable a reduction in absorbance. In the case of *Camellia sinensis*, a dramatic increase in nanoparticle synthesis was observed versus using previous method (boiling water extract), but the rate of conversion to silver nanoparticles was less ( Figure 9 ). It might be interpreted that releasing reductive agents from the powder in the reaction mixture are dependent on the time span. By a gradual increase in amount of plant powder, the nanoparticle formation was increased. In the case of the boiled reaction mixture, the absorption stopped at 24 h. It could be argued that the reduction factors in the extract were limited after boiling. As presented in Figure 10 , the results of the absorbance spectra of Walnut and Tea (*C. sinensis*) powders were compared. Walnut consistently produced more nanoparticles than *C. sinensis*. The stability of both nanoparticles was similar.

### 4.7. Comparison of Three Methods Used for Nano-Silver Particle Formation

**Figure 11:**
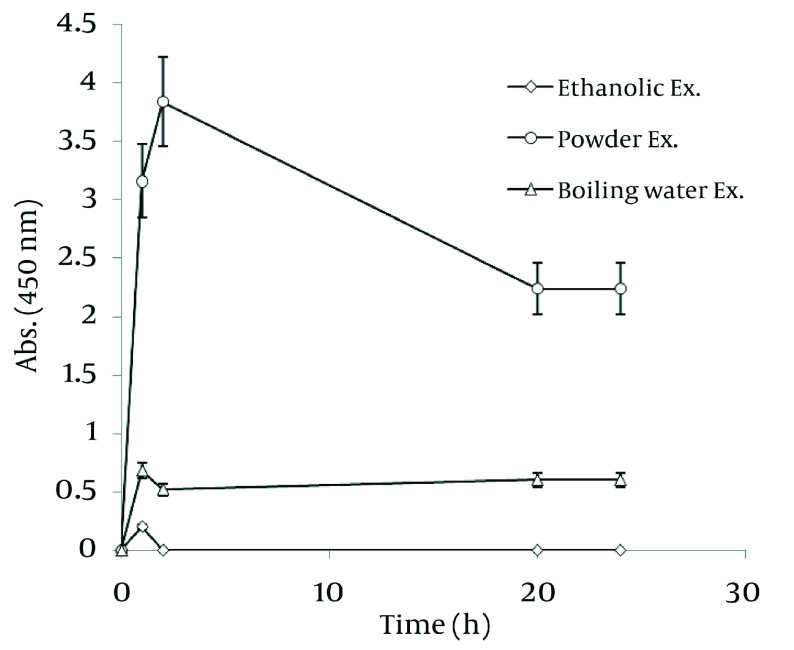
Comparison of Ag Nanoparticle Formation of Three Methods on *J. regia*

**Figure 12:**
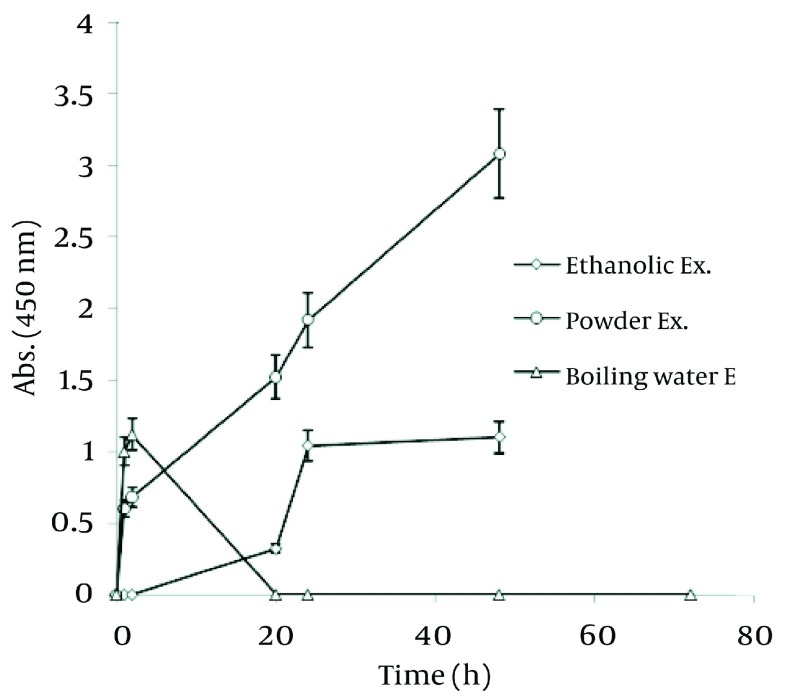
Comparison of Ag Nanoparticle Formation of Three Methods on *C. sinensis*

In order to realize the best route of employing Walnut leaf for Ag nanoparticle production, time intervals of sliver nanoparticle generation with three techniques of using Walnut leaf were revealed in Figure 11 . It should be noted that amount of *J. regia* powder used in these three methods was the same. Utilizing Walnut powder yielded much higher nanoparticles than two other methods, followed by ethanolic extract and boiling water extract. The stability of generated nanoparticles was similar in all of methods. In the case of *C. sinensis*, using the plant powder was also the most effective. Reaction rate of using boiling water extract was higher than powder and the absorbance increased in the first hour, then immediately declined ( Figure 12 ).

### 4.8. Analysis of Silver Nanoparticles with Transmission Electron Microscope (TEM)

**Figure 13:**
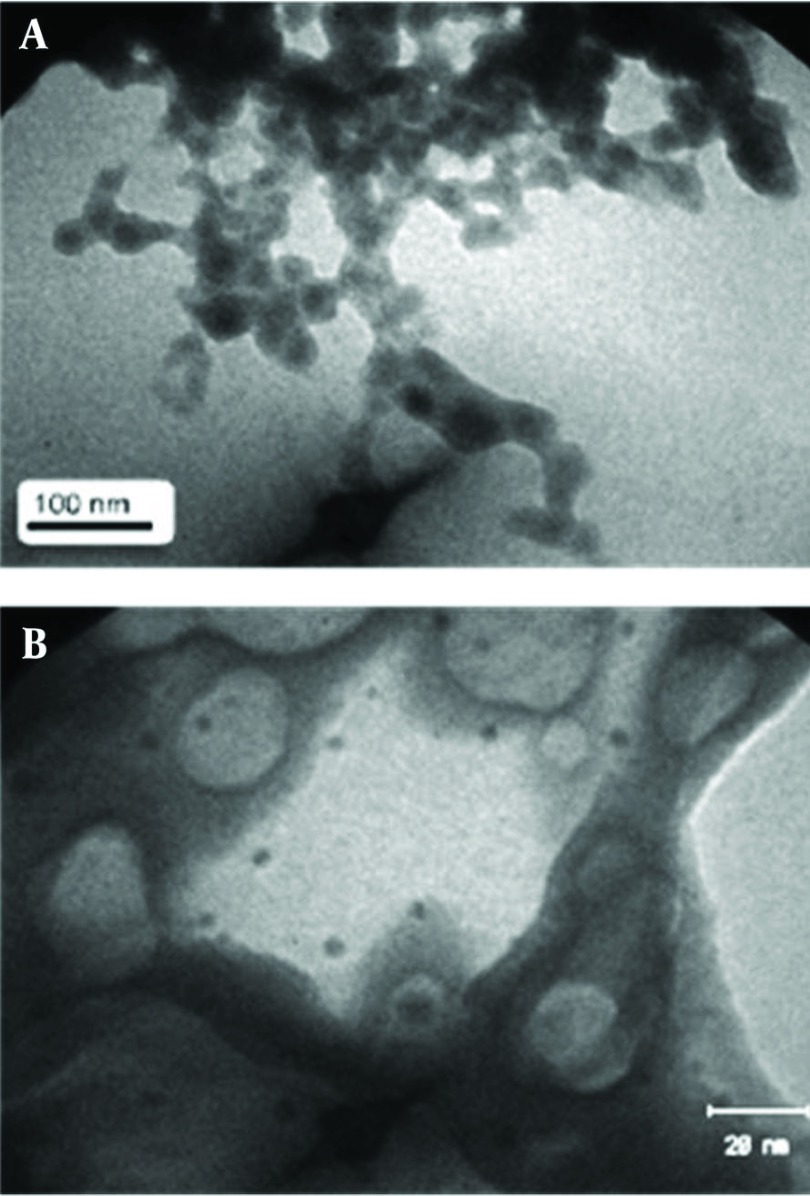
(A) TEM Micrographs Recorded From a Drop-coated Film of an Aqueous Solution of Ag+ ions Incubated With *J. regia* Water Extract. (B) *J. regia* Ethanolic Extract

Figure 13-A shows TEM micrograph of Ag nanoparticles produced by the reaction of AgNO_3_ solution with *J. regia* leaf powder, 2 hours following the start of the reaction. Silver nanoparticles were quasi- spherical, polydisperse (10-50 nm) and in aggregated form. Similar studies have been reported in Ag nanoparticle synthesis based on using plant powder. Bark powder of Cinnamon zeylanicum generated monodisperse Ag nanoparticles (31-40 nm) with spherical and rod shapes and in aggregated form (24), Euphorbia hirta produced cubic aggregated polydisperse silver nanoparticles in size of 40-50 nm (25) and using Cinnamomum camphora aggregated polydisperse nanoparticles (55-80 nm) with different shapes were synthesized (23), but in comparison with them, *J. regia* leaf powder yielded smaller dimensions of silver nanoparticles. Figure 13-B shows TEM micrograph of Ag nanoparticles produced by *J. regia* leaf ethanolic extract after 20 h. The nanoparticles were spherical, single (1-5 nm) and nearly monodisperse. The globules, which are seen in the graph, might be the fat molecules have been dissolved in the plant extract during ethanolic extraction and then after the addition of buffer and aqueous solutions, they were separated from the plant extract. Similar results based on using indirectly heated ethanolic extracts of Bryophyllum, Cyprus and Hydrilla was reported by Jha et al. and Fcc monodisperse Ag nanoparticles (2-5 nm) with no aggregation were produced (17).

## 5. Discussion

In the present study a new plant–*Juglans regia* debuted with an efficient production of silver nanoparticles. Moreover, different methods of employing this plant were compared for the first time. These trials illustrated that using the direct powder of Walnut produced more silver nanoparticles which is desirable from an economical and industrial perspective. However, using ethanolic extract of walnut leaf yielded non-aggregated nanoparticles with smaller dimensions. More details about its productivity were revealed, i.e. the influence of different extract concentrations and the effect of boiling the extract.

## References

[1] Dubey M, Bhadauri S (2009). Green synthesis of nanosilver particles from extract of Eucalyptus hybrida (safeda) leaf.. Dig J Nanomater Bios..

[2] Safaepour M, Shahverdi A, Shahverdi H, Khorramizadeh M, Gohari A (2009). Green synthesis of small silver nanoparticles using geraniol and its cytotoxicity against fibrosarcoma-wehi 164.. Avicenna J Med Biotechnol..

[3] Tripathy A, Raichur A, Chandrasekaran N, Prathna T, Mukherjee A (2010). Process variables in biomimetic synthesis of silver nanoparticles by aqueous extract of Azadirachta indica (Neem) leaves.. JNR..

[4] Wang Y, He X, Wang K, Zhang X, Tan W (2009). Barbated Skullcup herb extract-mediated biosynthesis of gold nanoparticles and its primary application in electrochemistry.. Colloids Surf..

[5] Shankar SS, Rai A, Ahmad A, Sastry M (2004). Rapid synthesis of Au, Ag, and bimetallic Au core-Ag shell nanoparticles using Neem (Azadirachta indica) leaf broth.. J Colloid Interface Sci..

[6] Song J, Kim B (2009). Rapid biological synthesis of silver nanoparticles using plant leaf extracts.. Bioprocess Biosyst Eng..

[7] Shankar S, Ahmad A, Sastry M (2003). Geranium leaf assisted biosynthesis of silver nanoparticles.. Biotechnol Prog..

[8] Chandran S, Chaudhary M, Pasricha R, Ahmad A, Sastry M (2006). Synthesis of gold nanotriangles and silver nanoparticles using Aloe vera plant extract.. Biotechnol Prog..

[9] Begum N, Mondal S, Basu S, Laskar R, Mandal D (2009). Biogenic synthesis of Au and Ag nanoparticles using aqueous solutions of Black Tea leaf extracts.. Colloids Surf..

[10] Jain D, Kumar Danima H, Kachhwaha S, SL. K (2009). Synthesis of plant- mediated silver nanoparticles using Papaya fruit extract and evaluation of their antimicrobial activites.. Dig J Nanomater Bios..

[11] Rajasekharreddy P, Usha Rani P, Sreedhar B (2010). Qualitative assessment of silver and gold nanoparticle synthesis in various plants: a photobiological approach.. JNR..

[12] Philip D (2010). Green synthesis of gold and silver nanoparticles using Hibiscus rosa sinensis.. Physica E..

[13] Sathyavathi R, Krishna M, Rao S, Saritha R, Rao D (2010). Biosynthesis of Silver Nanoparticles Using Coriandrum Sativum Leaf Extract and Their Application in Nonlinear Optics.. Ad Sci Letters..

[14] Parashar V, Parashar R, Sharma B, Pandey A (2009). Parthenium leaf extract mediated synthesis of silver nanoparticles: a novel approach towards weed utilization.. Dig J Nanomater Bios..

[15] Jha A, Prasad K, Kumar V (2009). Biosynthesis of silver nanoparticles using Eclipta leaf.. Biotechnol Prog..

[16] Ankanna S, Prasad T, Elumalai E, Savithramma N (2010). Production of biogenic silver nanoparticles using Boswellia ovalifoliolata stem bark.. Dig J Nanomater Bios..

[17] Jha A, Prasad K, Kulkarni A (2009). Plant system: nature's nanofactory.. Colloids Surf..

[18] Song J, Kim B (2008). Biological synthesis of bimetallic Au/Ag nanoparticles using Persimmon (Diopyros kaki) leaf extract.. Korean J Chem Engin..

[19] Li S, Shen Y, Xie A, Yu X, Qiu L, Zhang L (2007). Green synthesis of silver nanoparticles using Capsicum annuum L. extract.. Green Chemistry..

[20] Parashar U, Saxena P, Srivastava A (2009). Bioinspired synthesis of silver nanoparticles.. Dig J Nanomater Bios..

[21] Vilchis-Nestor A, Sánchez-Mendieta V, Camacho-López M, Gómez-Espinosa R, Camacho-López M, Arenas-Alatorre J (2008). Solventless synthesis and optical properties of Au and Ag nanoparticles using Camellia sinensis extract.. MATL..

[22] Kesharwani J, Yoon K, Hwang J, Rai M (2009). Phytofabrication of Silver Nanoparticles by Leaf Extract of Datura metel: Hypothetical Mechanism Involved in Synthesis.. J Bionanoscience..

[23] Huang J, Li Q, Sun D, Y. L, Y. S, X. Y (2007). Biosynthesis of silver and gold nanoparticles by novel sundried Cinnamomum camphora leaf.. Nanotechnology..

[24] Sathishkumar M, Sneha K, Won SW, Cho CW, Kim S, Yun YS (2009). Cinnamon zeylanicum bark extract and powder mediated green synthesis of nano-crystalline silver particles and its bactericidal activity.. Colloids Surf..

[25] Elumalai EK, Prasad T, Hemachandran J, Therasa S, Thirumalai T, David E (2010). Extracellular synthesis of silver nanoparticles using leaves of Euphorbia hirta and their antibacterial activities.. J Pharm Sci..

[26] Geethalakshmi R, Sarada D (2010). Synthesis of plant-mediated silver nanoparticles using Trianthema decandra extract and evaluation of their anti microbial activities.. Int J Engin Sci Technol..

[27] Fierascu R, Ion R, Dumitriu I (2010). Noble metals nanoparticles synthesis in plant extracts, Optoelectronics and Advanced Materials.. Rapid Communications..

[28] Mallikarjuna K, Narasimha G, Dillip G, Praveen B, B. S, Lakshmi C (2011). Green synthesis of silver nanoparticles using Ocimmum leaf extract and their characterization.. Dig J Nanomater Bios..

[29] Prasad K, Pathak D, Patel A, Dalwadi P, Prasad R, Patel P (2011). Biogenic synthesis of silver nanoparticles using Nicotiana tobaccum leaf extract and study of their antibacterial effect.. Afr J Biotechnol..

[30] Mukunthan K, Elumalai E, Patel T, Murty V (2011). Catharanthus roseus: a natural source for the synthesis of silver nanoparticles.. Asian Pac J Trop Biomed..

[31] Prasad T, Elumalai E (2011). Biofabrication of Ag nanoparticles using Moringa oleifera leaf extract and their antimicrobial activity.. Asian Pac J Trop Biomed..

[32] Kaviya S, Santhanalakshmi J, Viswanathan B (2011). Green Synthesis of Silver Nanoparticles Using Polyalthia longifolia Leaf Extract along with D-Sorbitol: Study of Antibacterial Activity. J Nanotechnology..

[33] Mishra A, Bhadauria S, Gaur M, Pasricha R, Kushwah B (2010). Synthesis of Gold Nanoparticles by Leaves of Zero-Calorie Sweetener Herb (Stevia rebaudiana) and Their Nanoscopic Characterization by Spectroscopy and Microscopy.. Intel J Green Nanotech..

